# A Rare Case of Persistent COVID-19 Infection With Aspergillosis in a 12-Year-Old Child

**DOI:** 10.7759/cureus.33973

**Published:** 2023-01-19

**Authors:** Rasagnya M Reddy, Amar Taksande, Mahaveer S Lakra, Mayur B Wanjari

**Affiliations:** 1 Department of Pediatrics, Jawaharlal Nehru Medical College, Datta Meghe Institute of Higher Education & Research, Wardha, IND; 2 Department of Research and Development, Jawaharlal Nehru Medical College, Datta Meghe Institute of Higher Education & Research, Wardha, IND

**Keywords:** sars-cov-2, covid-19 in children, prolonged viral shedding, aspergillosis, genomic integration

## Abstract

At the end of 2019, coronavirus disease 2019 (COVID-19) was first detected in Wuhan. In March 2020, COVID-19 became a global pandemic. Saudi Arabia registered the first case of COVID-19 on March 2, 2020. COVID-19 can affect any organ in the body. It affects the respiratory system predominantly. Reverse transcriptase-polymerase chain reaction (RT-PCR) is used to diagnose COVID-19, and the preferred swab is the nasopharyngeal swab. The shedding of the virus continues for about 20 days after the onset of the symptoms. There may be prolonged shedding in a few cases without any symptoms. Viral cultures are used for the confirmation of the shedding. Although the preferred mode of diagnosis is RT-PCR, enzyme-linked immunosorbent assay helps in the diagnosis of antibodies. Here, we report a rare case of prolonged viral shedding for more than 14 weeks. The patient had a prolonged COVID-19 infection, which caused immunosuppression, following which the patient presented with an infection.

## Introduction

Coronavirus disease 2019 (COVID-19) is a predominantly respiratory system-affecting, multi-organ disease. The preferred test is reverse transcriptase-polymerase chain reaction (RT-PCR) via a nasopharyngeal or throat swab sample, which detects the viral RNA [1]. From the onset of symptoms, 12-20 days is the median duration for the shedding of severe acute respiratory syndrome coronavirus 2 (SARS-CoV-2). Recent studies have shown that SARS-CoV-2 can be shed by asymptomatic patients for weeks or even longer. Risk factors for prolonged viral shedding include delayed admission to the hospital, severe illness at admission, administration of corticosteroids, immunoglobulins, and male gender [2].

Long-term sequelae and complications can be seen in COVID-19 patients. The exact prevalence and risk factors for long-term sequelae and complications are yet to be elucidated. The sensitivities of the many different detection tests for COVID-19 vary. The preferred method for establishing the diagnosis is the detection of RNA by RT-PCR [3]. To identify immunity and past infections and for screening, an enzyme-linked immunosorbent assay (ELISA) is used, which detects immunoglobulin (Ig)G and IgM antibodies against the virus. Here, we report the case of a 12-year-old male child with COVID-19 with persistently positive SARS-CoV-2 on repeated RT-PCR nasopharyngeal swab testing, lasting 14 weeks after the initial diagnosis was made. This is a very unusual and rare association with SARS-CoV-2 infection.

## Case presentation

**Figure 1:**
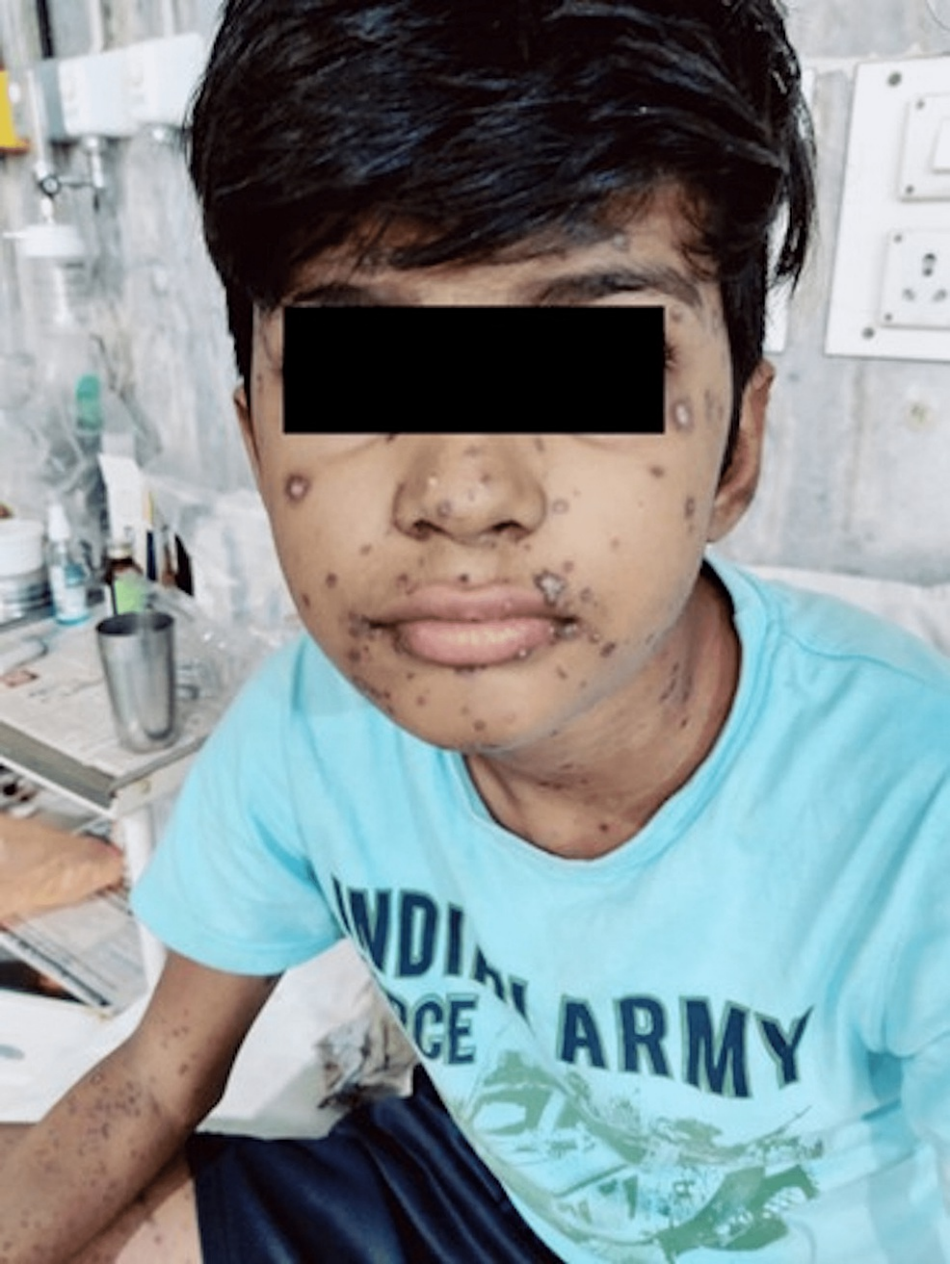
Multiple confluent vesicular rashes over the face, skin, and extremities in different stages.

A 12-year-old male child presented with complaints of fever and cough for five days. There was no history of vomiting, loose motion, pain in the abdomen, rashes, altered behavior, and cyanosis (Figure 1). The child was conscious, oriented, anicteric, and febrile with a pulse rate of 112 beats/minute, respiratory rate of 34 breaths/minute, blood pressure of 112/74 mmHg, which was in 90 centiles, and SpO_2_ of 94% on room air with no cyanosis or any respiratory distress. The patient was a known case of immune thrombocytopenia diagnosed two years back. The patient was treated with steroids for three months but was not compliant with the treatment. As he developed thrombocytopenia later, a steroid was started for the same.

On admission, the patient was vitally stable, and rapid antigen was done in casualty, which was positive. The patient was admitted to the presumptive ward, and RT-PCR was sent, which was positive. As the child was hemodynamically stable, symptomatic treatment was started. Laboratory investigations are shown in Table 1.

**Table 1:** Blood investigation findings of the patient.

Investigation	Patient value	Normal value
Hemoglobin (g/dL)	10.1	13.2–16.6
Total leucocyte count (/µL)	9,300	4,500–11,000
Platelet count (/µL)	138,000	150,000–450,000
D-dimer (ng/mL)	515	250
Erythrocyte sedimentation rate (mm/hour)	28	0–22
C-reactive protein (mg/L)	145	8–10
Ferritin (ng/mL)	348	24–336
Lactate dehydrogenase (U/L)	277	105–333
Random blood sugar (mg/dL)	105	Less than 140
Vitamin D (IU)	18	600

**Figure 2:**
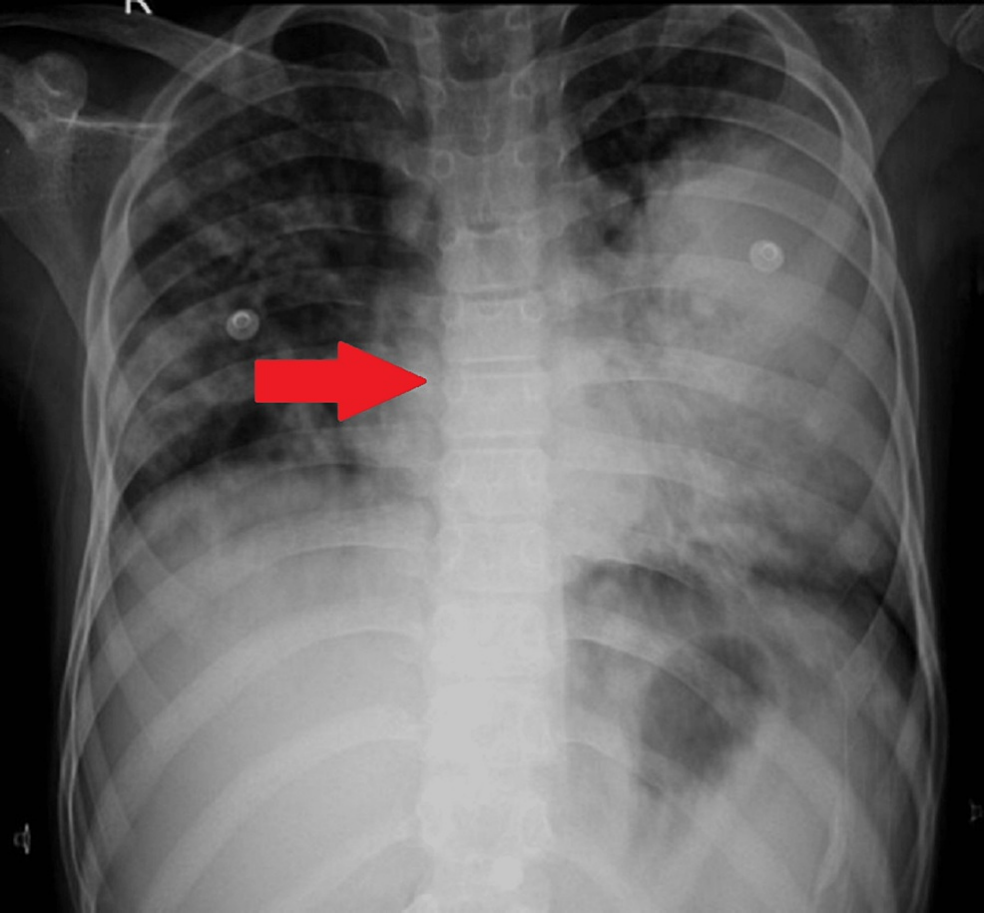
Chest X-ray showing homogenous opacity on the left side of the chest with mild haziness on the right side, suggestive of consolidation.

**Figure 3:**
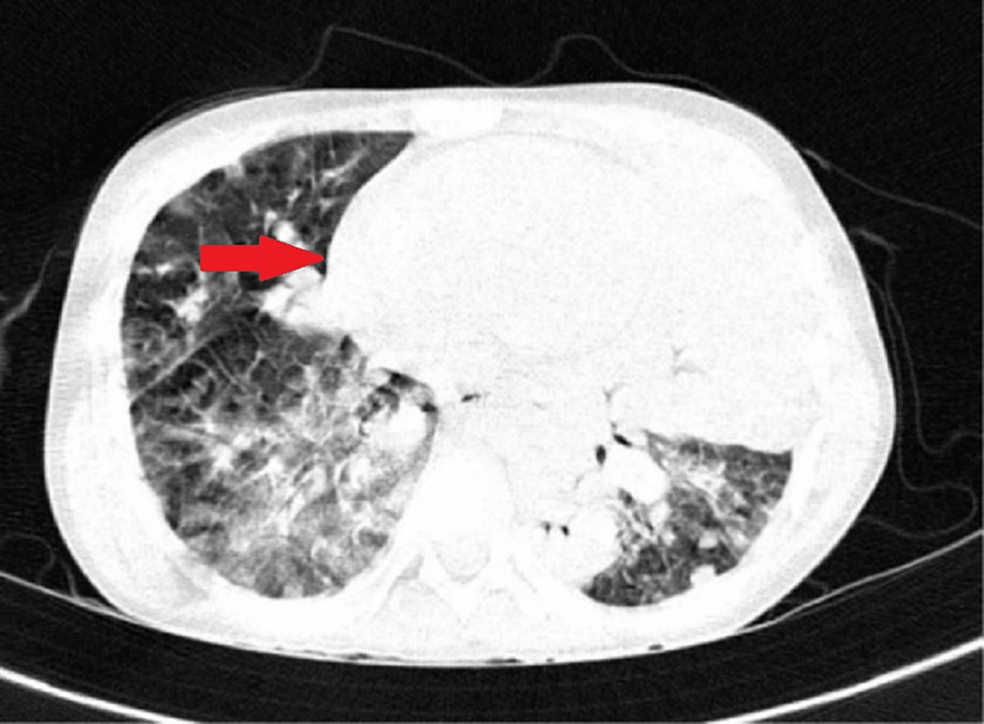
Multiple discrete and confluent patches of consolidation noted in bilateral lungs with a ball-like homogeneous opacity, suggestive of fungal balls.

A chest X-ray was done which was suggestive of consolidation (Figure 2). On day five of the hospital stay, the patient developed painful vesicular lesions over the entire body. It was diagnosed to be varicella zoster. The patient did not have a previous history of varicella and had received two doses of vaccination; one was taken at 15 months of age, and the second was taken at six years of age. Injection piperacillin, injection amikacin, and injection acyclovir were started. The patient developed persistent respiratory complaints. High-resolution computed tomography (HRCT) of the chest showed fluffy shadows suggestive of aspergillosis (Figure 3), and the sputum culture was positive for aspergillosis. Hence, injection amphotericin B was started, and a repeat RT-PCR was sent 14 days after the initial positive test.

Over time, vesicular lesions and fever spikes decreased, and the patient was symptom-free. Laboratory investigations such as complete blood count, liver function test, kidney function test, and inflammatory markers were within normal limits. The human immunodeficiency virus test was non-reactive. Repeat swabs were sent every 15 days, which again tested positive. The swabs were repeated every 15 days, but they were persistently positive for two months throughout the hospital stay. The child improved over time and was advised for home isolation and discharged at the parent’s request. The patient did not come for a follow-up for one and a half months after the discharge. Suddenly, he presented to the emergency department with complaints of fever for eight days and breathlessness and cough for three days. COVID-19 RT-PCR test was again found to be positive.

On examination, tachypnea was present, along with intercostal retractions and bilateral crepitation. As the patient was not maintaining oxygen saturation, he was put on oxygenby continuous positive airway pressure. Additionally, injection ceftriaxone and injection amikacin were started. Further investigations showed hemoglobin of 8 g/dL, total leukocyte count of 18,400/µL, platelet count of 405,000/µL, D-dimer of 908 ng/mL, C-reactive protein of 239 mg/L, erythrocyte sedimentation rate of 110 mm/hour, and ferritin of 505 ng/mL. Chest X-ray showed a patch over the left side of the lung. HRCT of the thorax showed multiple discrete and confluent patches of consolidation noted in bilateral lungs, as shown in Figure 3. The patient was referred to a higher center for further management according to COVID-19 protocols.

## Discussion

The structural proteins of coronaviruses include nucleocapsid and spike proteins and envelope [4]. An open reading frame called ORF1ab is responsible for their replication and transcription, and RNA is dependent on the RNA polymerase gene (RdRp). RT-PCR uses ORF1ab, E, RdRp, N, and S genes as targets to detect SARS-CoV-2 [5]. An upper respiratory tract PCR sample may detect SARS-CoV-2 even a few days before the onset of symptoms. Viral shedding persists for a varying number of days after the onset of symptoms, but a study reported a median duration of 11 days [6]. Even if the nasopharyngeal swabs of infected patients are negative, newer evidence suggests the long-term shedding of SARS-CoV-2 in the urine and stool of infected patients [7]. Fever (>38.5°C), severe disease, male gender, concomitant hypertension, steroid use, invasive ventilation, intensive care unit admission, and lack of antiviral drugs are the factors independently associated with prolonged respiratory viral shedding [8]. The presentation of COVID-19 infection in children is different from adults. Children are less severely affected because of innate immunity and less presentation of angiotensin-converting enzyme receptors on their epithelial cells. Most children are asymptomatic, do not require aggressive treatment or oxygen therapy, and rarely need invasive ventilation [9].

Three putative pathways, including reinfection, leftover viral RNA fragments, and genomic integration, have been proposed for the persistence of pathogenicity of SARS-CoV-2 infection. Uncertainty regarding the interaction between the immune system and SARS-CoV-2 infection is reflected in the etiological mechanism underlying persistent RNA shedding after recovery [10]. Reinfection appears to be the only situation with a significant risk of severe infection and virus dissemination, necessitating a high level of clinical suspicion, especially in those who present with symptoms after or more than 90 days after contacting SARS-CoV-2.

Similar cases have also been reported by Bullard et al. [11] who described the infectiousness through viral cultures which were viable when the RT-PCR cycle threshold (CT) value was <24 within less than eight days from symptom onset when SARS-CoV-2 was cultured. Nonetheless, it is not necessarily indicated by RT-PCR positivity that an individual is infectious or is shedding live viruses. The optimal CT cut-off value is unknown. Our patient was persistently COVID-19 positive for 14 weeks and was symptomatic on and off with various presentations such as aspergillosis, mucormycosis, acute respiratory distress syndrome, and varicella infection. Such persistent shedding of the COVID-19 virus with all manifestations is rare.

## Conclusions

Persistent shedding of the virus in COVID-19 is rare but can be encountered in children as well. It should be differentiated from reinfection and persistence or chronicity of the same infection. If such persistent viral shedding is present, the patient may be isolated, and home quarantine should be followed. In view of the current pandemic, every physician should be aware of this new presentation of COVID-19 infection in children.
